# Junín Virus Infection of Human Hematopoietic Progenitors Impairs *In Vitro* Proplatelet Formation and Platelet Release via a Bystander Effect Involving Type I IFN Signaling

**DOI:** 10.1371/journal.ppat.1000847

**Published:** 2010-04-15

**Authors:** Roberto G. Pozner, Agustín E. Ure, Carolina Jaquenod de Giusti, Lina P. D'Atri, Joseph E. Italiano, Oscar Torres, Victor Romanowski, Mirta Schattner, Ricardo M. Gómez

**Affiliations:** 1 Department of Thrombosis and Hemostasis, Hematological Research Institute “Mariano R Castex”, National Academy of Medicine, CONICET, Buenos Aires, Argentina; 2 Biotechnology and Molecular Biology Institute, CONICET-UNLP, La Plata, Argentina; 3 Division of Translational Medicine, Brigham and Women's Hospital, Boston, Massachusetts, United States of America; 4 Department of Vascular Biology, Children's Hospital Boston, Boston, Massachusetts, United States of America; North Carolina State University, United States of America

## Abstract

Argentine hemorrhagic fever (AHF) is an endemo-epidemic disease caused by Junín virus (JUNV), a member of the *arenaviridae* family. Although a recently introduced live attenuated vaccine has proven to be effective, AHF remains a potentially lethal infection. Like in other viral hemorrhagic fevers (VHF), AHF patients present with fever and hemorrhagic complications. Although the causes of the bleeding are poorly understood, impaired hemostasis, endothelial cell dysfunction and low platelet counts have been described. Thrombocytopenia is a common feature in VHF syndromes, and it is a major sign for its diagnosis. However, the underlying pathogenic mechanism has not yet been elucidated. We hypothesized that thrombocytopenia results from a viral-triggered alteration of the megakaryo/thrombopoiesis process. Therefore, we evaluated the impact of JUNV on megakaryopoiesis using an *in vitro* model of human CD34^+^ cells stimulated with thrombopoietin. Our results showed that CD34^+^ cells are infected with JUNV in a restricted fashion. Infection was transferrin receptor 1 (TfR1)-dependent and the surface expression of TfR1 was higher in infected cultures, suggesting a novel arenaviral dissemination strategy in hematopoietic progenitor cells. Although proliferation, survival, and commitment in JUNV-infected cultures were normal, viral infection impaired thrombopoiesis by decreasing *in vitro* proplatelet formation, platelet release, and P-selectin externalization via a bystander effect. The decrease in platelet release was also TfR1-dependent, mimicked by poly(I:C), and type I interferon (IFN α/β) was implicated as a key paracrine mediator. Among the relevant molecules studied, only the transcription factor NF-E2 showed a moderate decrease in expression in megakaryocytes from either infected cultures or after type I IFN treatment. Moreover, type I IFN-treated megakaryocytes presented ultrastructural abnormalities resembling the reported thrombocytopenic NF-E2^−/−^ mouse phenotype. Our study introduces a potential mechanism for thrombocytopenia in VHF and other diseases associated with increased bone marrow type I IFN levels.

## Author Summary

Argentine hemorrhagic fever (AHF) is an endemo-epidemic disease caused by Junín virus (JUNV). Although a recently introduced live attenuated vaccine has proven to be effective, AHF remains a potentially lethal infection and JUNV is considered to be a potential biological weapon. Like other viral hemorrhagic fevers (VHF), AHF patients present fever with a combination of neurological and bleeding complications. Although the causes of the bleeding are poorly understood, impaired hemostasis and endothelial cell function as well as low platelet counts have been described. In this study, we have examined the impact of JUNV on an *in vitro* model of platelet production. We found that neither infection of hematopoietic progenitors with JUNV nor poly(I:C) (a double-stranded RNA that mimics viral infection) affected cell survival or megakaryocyte generation. However, these treatments triggered the main anti-viral cytokines produced by host type I IFN (IFN α/β), which acted in a paracrine fashion and led to abnormal platelet formation. Thus, this study identifies type I IFN as a new regulator that selectively affects the last steps of megakaryocyte lifespan, and it suggests a potential mechanism for thrombocytopenia in AHF and other diseases associated with increased bone marrow type I IFN levels.

## Introduction

Viral hemorrhagic fever (VHF) is an acute systemic febrile syndrome caused by a diverse group of RNA viruses from the *Arenaviridae*, *Bunyaviridae*, *Filoviridae*, and *Flaviviridae* viral families [1]–[3]. Patients with this syndrome present with a combination of fever, prostration, malaise, and differing degrees of hematological complications [1], [4]. When severe, patients with VHF can present with generalized bleeding that results from alterations of the vascular endothelium, blood coagulation components and platelet levels [5]–[8]. Moreover, a plasma platelet aggregation inhibitor has been described in patients with different types of VHF [9], [10]. These findings suggest that different etiologic agents of VHF may share common processes for disturbing hemostasis during infection, leading to a systemic and frequently fatal disease. In fact, thrombocytopenia is one of the most consistent findings among human patients and experimental animal models of VHF, and it is used as a major diagnostic feature in patients with VHF [11], [12]. However, the causes of the thrombocytopenia associated with VHF remain poorly understood. In this connection, disseminated intravascular coagulation (DIC) could explain platelet consumption; nevertheless, the occurrence of DIC in VHF infections is inconclusive, at least for the arenavirus family [12]. Therefore, a high level of splenic sequestration or impaired megakaryo/thrombopoiesis could be the major physiopathogenic mechanisms responsible for the low platelet count.

We hypothesized that the thrombocytopenia observed in VHF is the result of a viral-triggered alteration of the megakaryo/thrombopoiesis process. Support for this hypothesis comes from studies of experimental murine lymphocytic choriomeningitis virus (LCMV) infection showing an association between thrombocytopenia and reduced megakaryocyte number at the bone marrow level [13] and the observation that the reversible depression of hematopoiesis during early LCMV infection is a direct effect of IFN α/β [14]. In addition, megakaryocyte alterations were found in patients with Argentine hemorrhagic fever (AHF) [15] and Dengue [16], and in experimental studies involving Junín virus (JUNV) infection of guinea pigs [17] and the primates *Callithrix jacchus* [18] and *Rhesus macaques* [19].

AHF is an endemo-epidemic disease caused by JUNV, a member of the *Arenaviridae* family. Although a recently introduced live attenuated vaccine has proven to be effective, AHF remains a potentially lethal infection and JUNV is considered to be a potential biological weapon [20]. Relative to other etiologic agents of VHF, JUNV is a suitable model for studying VHF pathogenesis due to the availability of a relatively large amount of data from both human and experimental infections, viral strains with known nucleotide sequences, several monoclonal antibodies against various components of the virus and an effective protective vaccine [21]–[23].

In this study, we evaluated whether the different megakaryopoiesi/thrombopoiesis stages (including proliferation, commitment, maturation, proplatelet formation and platelet release and function) are affected by JUNV infection of hematopoietic progenitor cells. Our results showed that human CD34^+^ cells are infected by JUNV. Furthermore, we have identified selective inhibition of platelet release involving transferrin receptor 1 (TfR1) and type I IFN (IFN α/β) as paracrine mediators.

## Results

### An *in vitro* model of megakaryopoiesis

**Figure 1:**
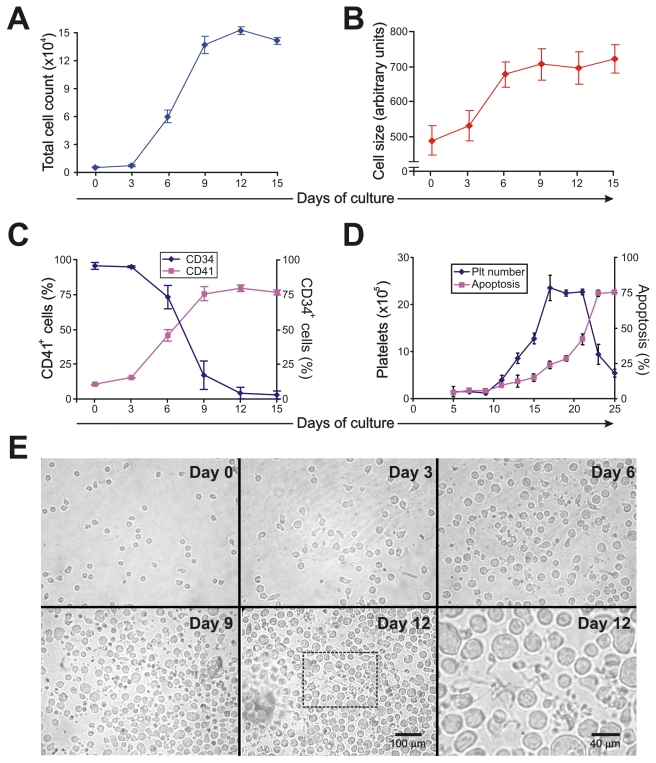
Characterization of liquid cultures of human CD34^+^ cells stimulated by TPO. CD34^+^ cells (1×10^4^/ml) purified by immunomagnetic positive selection were cultured in IMDM containing 5% human serum and TPO (25 ng/ml added at days one and seven of the cell culture). At the indicated culture times, (A) total cell count was determined with a hemocytometer and (B) cell size was analyzed by flow cytometry. (C) CD41 and CD34 expression were evaluated by labeling cells with specific mAbs or corresponding matched isotypes and establishing the percentage of positive cells by flow cytometry analysis. (D) Platelet (Plt) count was evaluated by flow cytometry and cellular apoptosis was determined by detecting nuclear morphological changes of cells stained with acridine orange and ethidium bromide by fluorescence microscopy. (E) Culture morphology was assessed by phase contrast microscopy [original magnification 450×, except day 12 inset (1200×)]. The values expressed in panels A–D represent the mean ± SEM of five independent experiments. Panel E shows a representative experiment of five similar replicates.

To analyze whether the thrombocytopenia observed in AHF patients could be the result of altered megakaryopoiesis, we used an *in vitro* model of human CD34^+^ cells stimulated with thrombopoietin (TPO). Under our culture conditions, TPO stimulation promoted proliferation, an increase in cell size and CD41 expression and a decrease in CD34 expression (Figure 1A–C). The formation of long, branching processes called proplatelets, which showed small platelet-sized swellings that represented the developing platelets [24], was also observed on days 11–12 of culture (Figure 1E). In agreement with previous reports, platelets were released into the culture medium [25]. The kinetics of *in vitro* platelet release started at days 11–13, peaked at day 17 and declined after 21 days, correlating with the apoptosis/senescence of the cell culture (Figure 1D).

### JUNV infection selectively impairs *in vitro* platelet generation

**Figure 2:**
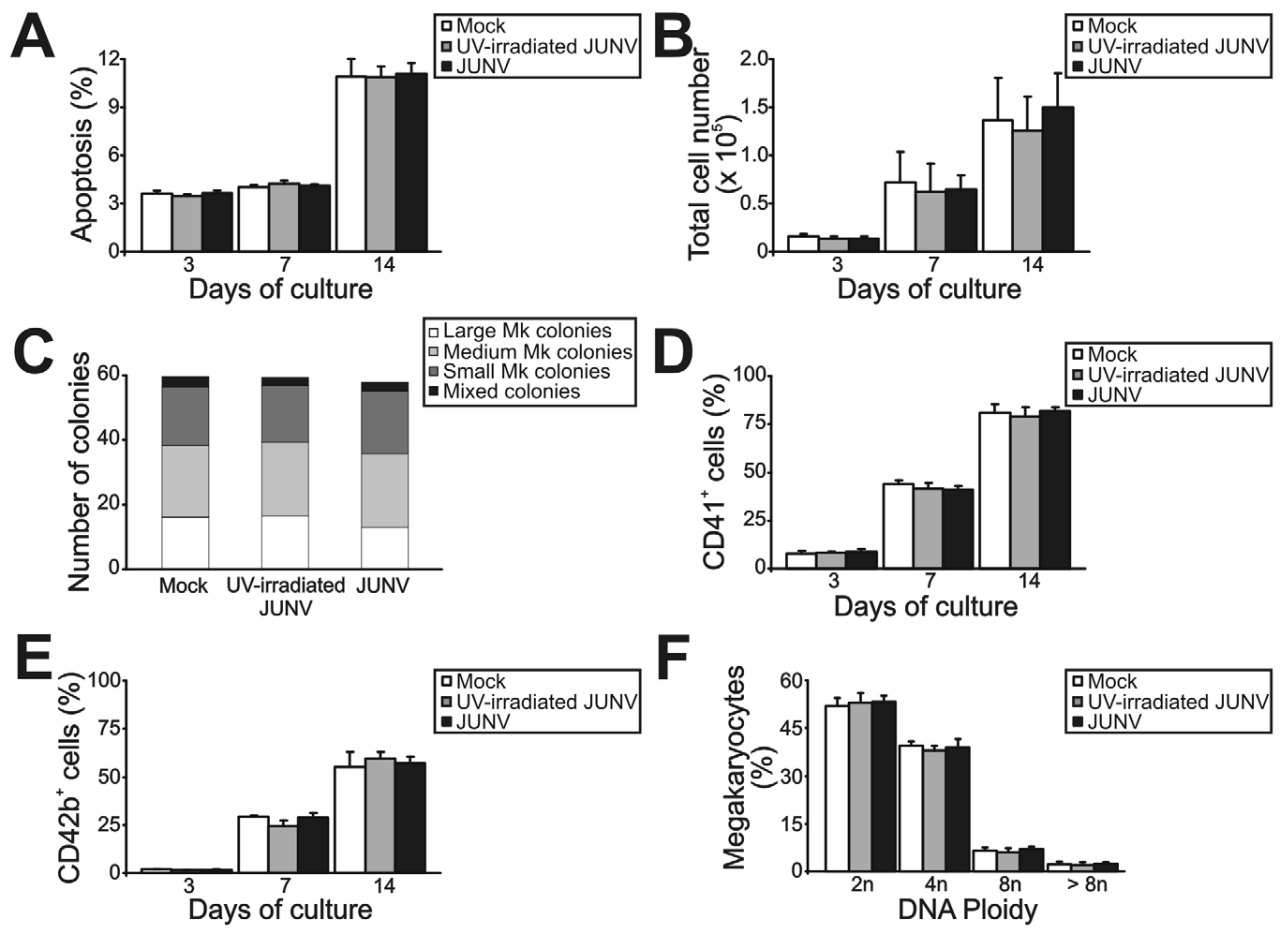
Influence of JUNV infection on cellular apoptosis, proliferation, clonogenic capacity and megakaryocyte development of TPO-stimulated CD34^+^ cells. CD34^+^ cells were infected with JUNV at a MOI of one or the equivalent volume of UV-irradiated virus or Vero cell supernatant (mock) for one hr at 37°C, washed, and then stimulated with TPO. (A) Apoptosis, (B) total cell count, (C) megakaryocyte colonies grown in collagen-based serum-free medium containing 50 ng/ml TPO, percentages of (D) CD41^+^ and (E) CD42b^+^ cells and (F) ploidy distribution were determined at the indicated days of culture, except for colonies and ploidy, which were counted after 12 or 14 days of culture, respectively. The values represent the mean ± SEM of four independent experiments.

**Figure 3:**
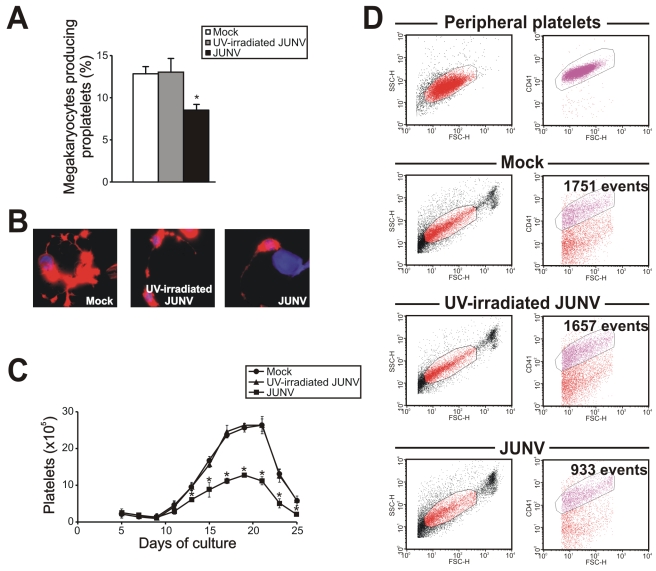
JUNV infection impairs proplatelet production and platelet release. CD34^+^ cells were mock-infected or infected with UV-irradiated JUNV or JUNV and then stimulated with TPO. (A) The number of megakaryocytes displaying proplatelets was determined at day 12 of the culture by F-actin and nuclear staining with phalloidin-TRITC and DAPI, respectively, and examined by fluorescence microscopy. The values represent the mean ± SEM of four independent experiments. (B) A representative picture of a proplatelet-displaying megakaryocyte. (C) Plt count was determined by flow cytometry. At different days, culture aliquots were stained with an anti-CD41-FITC mAb and the acquisition rate was fixed at 1 µl/sec for 100 sec. Events were collected without gating using a log scale for size (FSC) and intracellular granularity (SSC). An analytical gate was determined based on the scatter properties of peripheral blood platelets treated similarly. Culture-derived platelets were counted as CD41^+^ events with the same scatter properties as blood platelets. The values are expressed as platelets (×10^5^) and represent the mean ± SEM of six independent experiments. * indicates p<0.05 vs. UV-irradiated JUNV or mock. (D) A representative flow cytometric analysis 15 days after TPO stimulation.

Figure 2 shows that JUNV infection of CD34^+^ cells influenced neither their apoptosis, their proliferation rate, their maturation (CD42b^+^ and ploidy levels), megakaryocyte differentiation (CD41^+^) nor the clonogenic ability of megakaryocyte progenitors. Surprisingly, JUNV infection induced a significant decrease in both proplatelet formation (Figure 3A and B) and platelet release (Figure 3C and D) when compared to mock- or UV-irradiated JUNV-infected cultures. These results indicate that viral infection selectively hindered two key steps in thrombopoiesis that represent the last stage of the megakaryocyte lifespan, and viral replication was necessary to observe this effect.

To gain a deeper insight into the platelet biology of JUNV-infected cultures, we next explored the ability of platelets to express P-selectin. Our flow cytometric analysis showed that 11.1±3.3% of resting platelets generated in UV-irradiated JUNV-infected cultures expressed P-selectin on their surface, and after thrombin stimulation this rose to 49.2±4.1% (n = 3, p<0.05). In contrast, P-selectin externalization was significantly reduced in platelets derived from JUNV-infected cultures (3.3±1.3% and 13.5±4.1% for resting and thrombin-stimulated samples, respectively, n = 3).

### Susceptibility of CD34^+^ cells to JUNV infection

**Figure 4:**
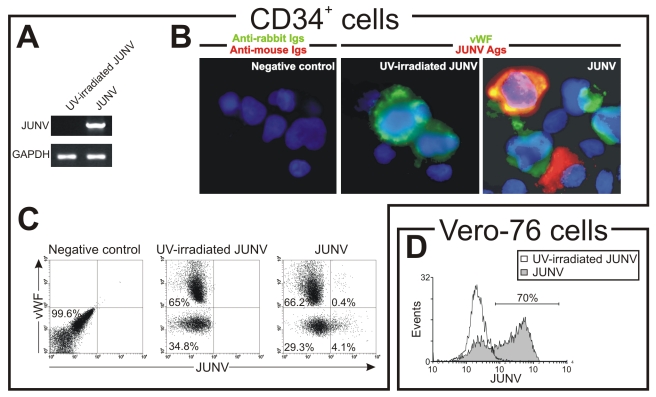
JUNV replication in CD34^+^ cells. CD34^+^ cells were inoculated with UV-irradiated JUNV or JUNV and stimulated with TPO for ten days. Viral replication was assayed by RT-PCR, immunofluorescence and flow cytometry. (A) RT-PCR studies. (B) To detect JUNV antigens by immunofluorescence cells were washed, cytocentrifuged on silanized glasses, fixed, permeabilized and incubated first with a pool of specific mAbs against JUNV and a rabbit-anti-human vWF polyclonal Ab to identify megakaryocytes, and then with FITC-conjugated anti-rabbit (green) and Cy3-conjugated anti-mouse Igs (red). The slides were counterstained with DAPI and photographed at 1000× magnification. (C) Cells were stained as in B and then analyzed by flow cytometry. (D) As a positive control, JUNV-susceptible Vero-76 cells were inoculated with JUNV and seven days later were stained with the pool of specific mAbs against JUNV followed by Cy3-anti-mouse Igs. Negative controls in B and C were performed by incubating cells only with secondary Abs. Panels show a representative experiment of three similar replicates.

As shown in Figure 4A, CD34^+^ cells were susceptible to JUNV infection. At ten days post-infection (p.i.), RNA from JUNV-inoculated CD34^+^ cells displayed a fragment of the viral genome, while RNA from cultures exposed to the UV-irradiated JUNV strain did not. Immunofluorescence and flow cytometric studies revealed that 4.1±0.2 and 4.9±0.5% of the cells were infected, respectively (Figure 4B and C). These results were in sharp contrast to the 68±11% of JUNV-positive cells observed after in-parallel infection of the susceptible Vero-76 cell line [26] (Figure 4D). Although some megakaryocytes were clearly positive for JUNV, most of the susceptible cells were not (Figure 4B and C). These results suggest that viral infection *per se* is not a direct cause of the observed reduced thrombopoiesis.

### Role of TfR1 in JUNV infection and impaired platelet production

**Figure 5:**
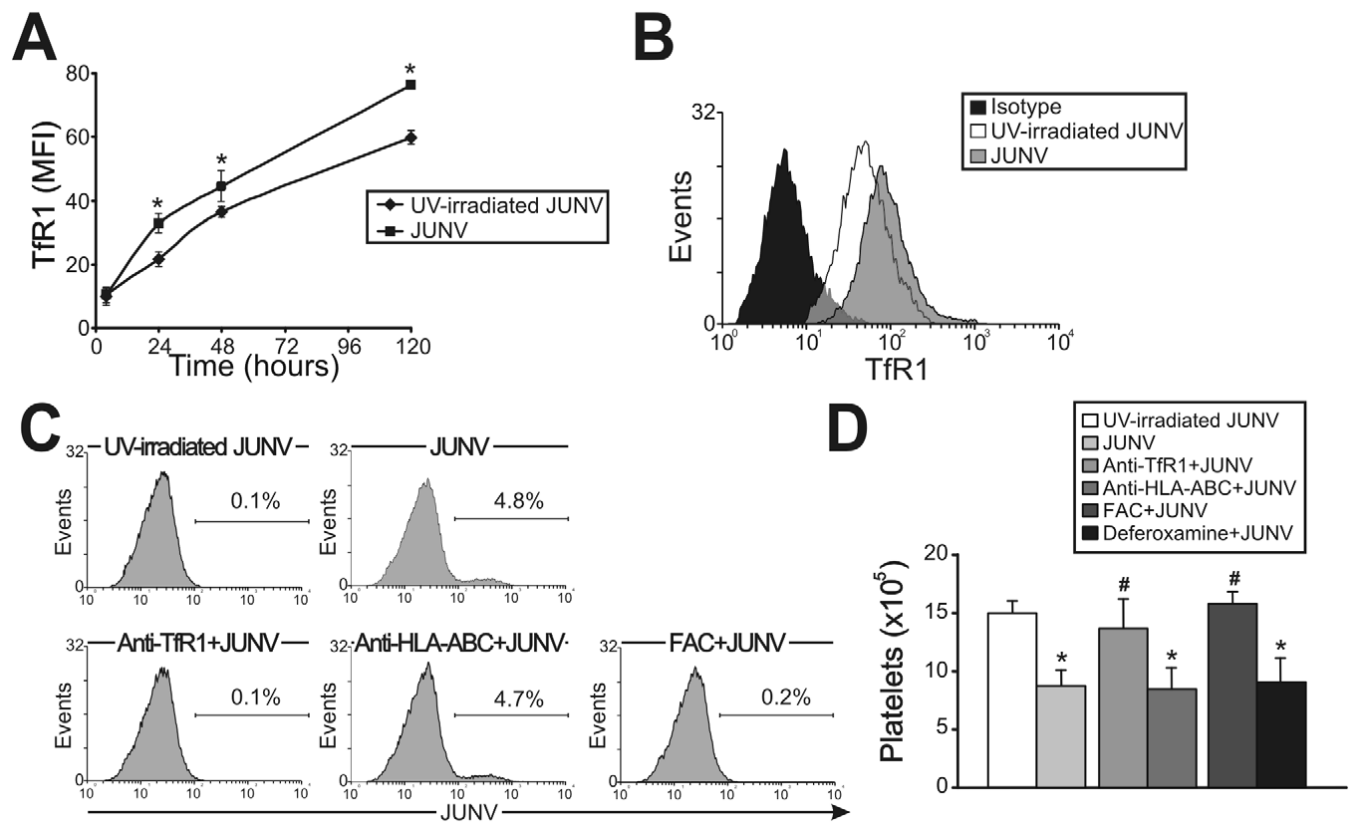
Role of TfR1 in JUNV infection and impaired platelet production. (A) The kinetics of TfR1 expression after JUNV infection in CD34^+^ cells stimulated with TPO were determined by flow cytometry. (B) Receptor expression was detected in UV-irradiated JUNV- and JUNV-infected cells incubated with FITC-anti-CD71 (anti-TfR1) mAb or with a matched isotype control. The histogram depicts a representative flow cytometric analysis of TfR1 staining after 120 hr of infection. (C) CD34^+^ cells were pre-incubated with an anti-CD71, anti-HLA-ABC mAb or ferric ammonium citrate (FAC, 10 µg/ml) for 1 hr (to down-regulate TfR1). Cells were then infected with JUNV and stimulated with TPO and viral antigens were detected by flow cytometry. The figure shows a representative experiment of three similar replicates. (D) CD34^+^ cells were treated as mentioned in C, and also with deferoxamine (1 µM) for 24 hr (to up-regulate TfR1). Platelets produced in culture were counted at day 15. The values represent the mean ± SEM of three independent experiments,* indicates p<0.05 vs. UV-irradiated JUNV, # indicates p<0.05 vs. JUNV.

In contrast to Lassa and other old world arenaviruses, which use α-dystroglycan to infect cells, it has been reported that TfR1 is the cellular receptor for several new world arenaviruses, including JUNV [27]. Therefore, we evaluated the expression of this protein in JUNV-infected cultures. Interestingly, TfR1 expression was significantly higher in JUNV-infected CD34^+^ cells compared to UV-irradiated JUNV-infected cells, suggesting that JUNV infection promotes further infection (Figure 5A and B).

Having demonstrated that TfR1 was up-regulated by viral infection, we next examined whether TfR1 has a role in JUNV infection and in the inhibitory effect of JUNV on platelet release. When CD34^+^ cells were incubated with a specific monoclonal antibody (mAb) against TfR1, neither viral antigens nor the inhibitory effect of JUNV on the number of platelets generated were observed (Figure 5C and D). In contrast, incubation with anti-HLA-ABC (an irrelevant mAb) had no effect. Similar results were obtained when TfR1 was first down-regulated by iron overload. However, up-regulation of TfR1 levels by iron deprivation did not modify the effect of JUNV on platelet generation (Figure 5D). These results not only unveil a potential new mechanism by which JUNV spreads its infection in hematopoietic progenitor cells, but they also provide evidence that the reduced thrombopoiesis is specifically related to JUNV infection.

### Involvement of the type I IFN pathway on JUNV-induced impairment of platelet release

**Figure 6:**
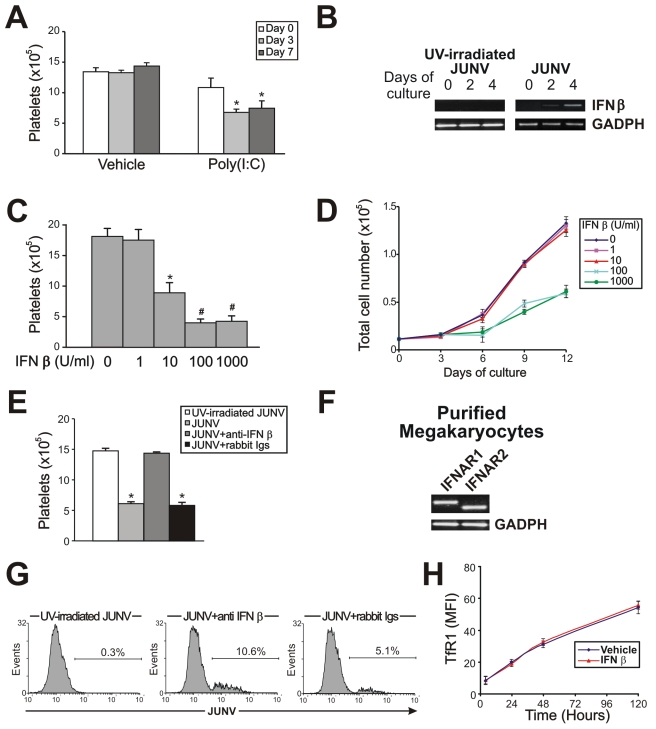
The role of the IFN β pathway in platelet production and JUNV infection. (A) CD34^+^ cells were treated with poly(I:C) (100 µg/ml) at the indicated days, stimulated with TPO, and Plt counts were determined at day 15 of the culture. The values represent the mean ± SEM of four independent experiments, * p<0.05 vs. vehicle (control). (B) IFN β mRNA levels in UV-irradiated JUNV- or JUNV-infected CD34^+^ cells were determined by RT-PCR at the indicated days of culture. The figure shows a representative experiment of three similar replicates. (C) CD34^+^ cells were treated with IFN β and stimulated with TPO. Plt counts were determined at day 15 of the culture. The values represent the mean ± SEM of three independent experiments,* indicates p<0.05 vs. no IFN β, # indicates p<0.05 vs. IFN β (10 U/ml). (D) Total cell number was determined at the indicated days of culture by counting cells with a hemocytometer. Similar results were obtained in MTT assays. The values represent the mean ± SEM of three independent experiments. (E) Anti-IFN β (1,000 neutralizing units) or an equal volume of rabbit Igs was added before CD34^+^ cell infection and Plt counts were determined at day 15. Values represent the mean ± SEM of three independent experiments, * indicates p<0.05 vs. UV-irradiated JUNV. (F) Type I IFN receptor subunit (IFNAR1 and 2) mRNAs were evaluated by RT-PCR in megakaryocyte precursors purified by immunomagnetic positive selection (99±1% of purity). The figure shows a representative experiment of three similar replicates. (G) CD34^+^ cells were infected with JUNV and 1000 neutralizing units of anti-IFN β or an equal volume of rabbit Igs were added before TPO stimulation. Viral antigens were detected by flow cytometry. The figure shows a representative experiment of two similar replicates. (H) The kinetics of TfR1 expression in the presence or absence (vehicle) of IFN β (10 U/ml) in CD34^+^ cells stimulated with TPO were determined by flow cytometry.

Because the number of infected cells was low and only a few were positively identified as megakaryocytes, it was reasonable to consider that the decrease in platelet release was most likely the result of a bystander cell effect rather than a direct effect of JUNV on megakaryocytes. Thus, to further study the biological effects of viral infection on platelet release, we analyzed the effect of poly(I:C), which mimics the double-stranded RNA (dsRNA) product from the replicative cycle of most viruses [28]. Figure 6A shows that the addition of poly(I:C) at early time points to TPO-stimulated CD34^+^ cell cultures significantly decreased platelet release. Similarly to JUNV infection, this inhibition was not related to apoptosis induction or a decrease in megakaryocyte generation (data not shown). These results suggest that the reduction in platelet release could be a more generalized effect and may not be restricted to JUNV infection.

Because poly(I:C) is a potent inducer of IFN β release *in vitro* and *in vivo* [29], we next tested if this was a functional mechanism in our *in vitro* system. While UV-irradiated JUNV-infected-CD34^+^ cell cultures stimulated by TPO did not express IFN β mRNA, increasing expression over time was observed in JUNV-infected cultures (Figure 6B). Accordingly, 10 U/ml of IFN β efficiently inhibited platelet release (Figure 6C). However, when used at concentrations of 100 U/ml or higher, it not only impaired platelet generation but also significantly decreased cell number (Figure 6D) without affecting the apoptosis rate (data not shown). These results suggest that IFN β might differentially regulate cellular responses, affecting only platelet production at low levels but affecting the cell cycle at higher levels. That IFN β was responsible for the JUNV-mediated biological effect was further supported by the observation that platelet production was restored to normal values in JUNV-infected cultures when IFN β was blocked by a neutralizing Ab (Figure 6E). To the best of our knowledge, the expression of type I IFN receptor in megakaryocytes has not been previously described. Thus, we examined the presence of mRNAs in purified megakaryocytes. RT-PCR studies revealed that megakaryocyte precursors transcribe both subunits of the IFN α/β receptor mRNAs (Figure 6F).

Because another general feature of the cells' response to viral infection is an increase in IFN α production, as a downstream product of the IFN β pathway, this molecule was also studied. Stimulation of CD34^+^ cells with IFN α mimicked the results obtained with IFN β (data not shown). These results were not surprising bearing in mind that both IFNs are ligands for the same receptor [30]. Overall, these results strongly indicate that the type I IFN signaling pathway may have a key role in the defective platelet release mediated by JUNV *in vitro*.

Considering that *in vivo* studies showed that megakaryocytes from type I IFN receptor knockout mice are more susceptible to LCMV infection [14], we next explored the role of type I IFN in JUNV infection. Figure 6G shows that the percentage of infected cells increased from 5% to 10% when the biological activity of IFN β was neutralized. However, recombinant IFN β did not modify TfR1 expression (Figure 6H) suggesting that viral replication in hematopoietic progenitor cells is controlled, at least in part, by IFN β production in a TfR1-independent manner.

### Mechanisms involved in JUNV inhibition of platelet formation

**Figure 7:**
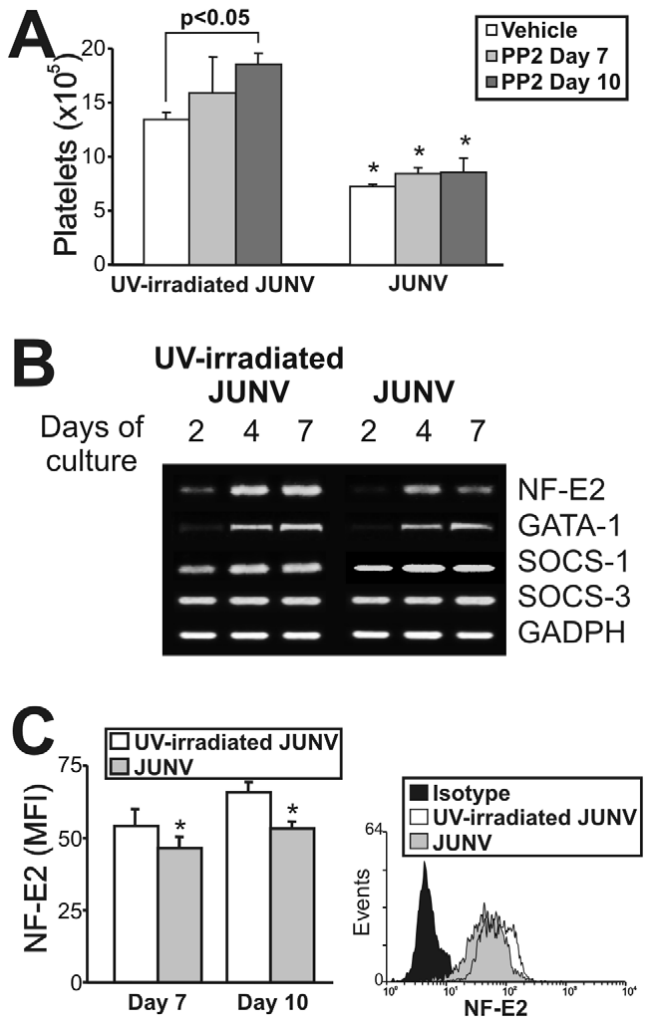
Intracellular mechanisms involved in the JUNV-induced inhibition of platelet production. CD34^+^ cells were UV-irradiated JUNV- or JUNV-infected, washed and stimulated with TPO. (A) The Src inhibitor PP2 (10 µM) was added at the indicated days, and Plt counts were determined at culture day 15. The values represent the mean ± SEM of four independent experiments, * indicates p<0.05 vs. UV-irradiated JUNV-treated cells. (B) Semi-quantitative RT-PCR analysis of relevant molecules involved in megakaryo/thrombopoiesis were performed at the indicated days of culture. The figure shows a representative experiment of three similar replicates. (C) NF-E2 expression was assessed in the megakaryocytic population by immunostaining the cells first with a PE-conjugated anti-CD41 mAb or an isotype-matched control. Then the cells were incubated with anti-NF-E2 polyclonal followed by FITC-conjugated swine anti-rabbit Igs. Cells were analyzed by flow cytometry. Non-specific fluorescence was assessed using rabbit serum instead of primary Ab. The values represent the mean ± SEM of three independent experiments,* indicates p<0.05 vs. UV-irradiated JUNV-infected cells. The histogram shows a representative flow cytometric analysis at day ten.

Although the signaling pathways regulating thrombopoiesis are still under study, Src kinases and several transcription factors have been shown to be involved as regulators of this process [31]. To clarify the mechanistic basis of the impaired platelet production in infected cultures, we first determined whether Src kinases were involved by using a specific inhibitor (PP2). Figure 7A shows that although Src inhibition enhanced platelet release in control cells, it did not prevent the JUNV-induced reduction in platelet number.

To examine the levels of relevant factors involved in megakaryo/thrombopoiesis, we performed a semi-quantitative RT-PCR assay. We found that the mRNAs of SOCS-1, SOCS-3 and GATA-1, three molecules involved in the initial steps of megakaryocyte development [32], were similar in JUNV- and UV-irradiated JUNV-infected cultures. In contrast, NF-E2, the main transcription factor regulating platelet biogenesis [33], was lower in JUNV-infected samples (Figure 7B). In addition, the total amount of NF-E2 protein was moderately but significantly diminished in the megakaryocytic population by viral infection (Figure 7C).

### Type I IFN decreases NF-E2 expression and induces intrinsic megakaryocytic abnormalities

**Figure 8:**
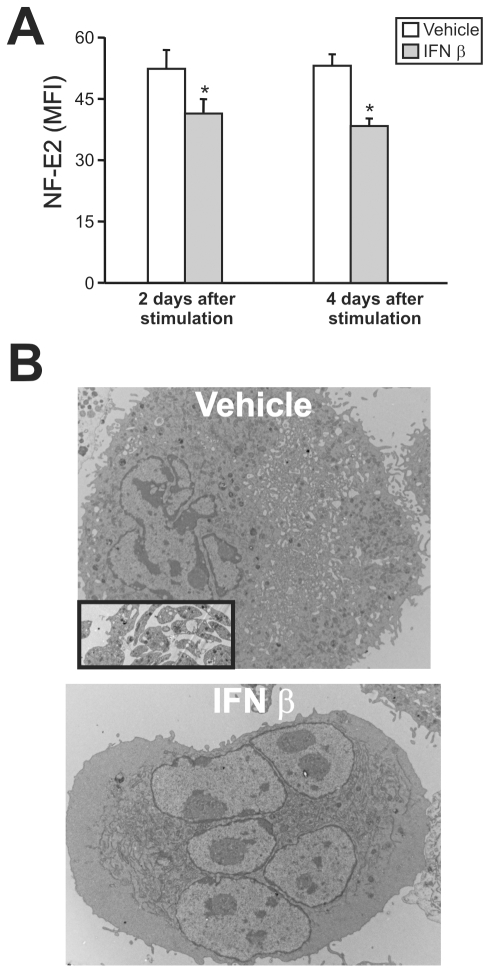
NF-E2 expression and ultrastructural studies of megakaryocytes treated with IFN β. (A) After 12 days of CD34^+^ cell TPO stimulation, megakaryocytes were purified by immunomagnetic positive selection (98±1% of purity) and NF-E2 expression was determined two or four days after IFN β (10 U/ml) treatment using the anti-NF-E2 polyclonal Ab (or rabbit serum) followed by FITC-conjugated swine anti-rabbit Igs. The values represent the mean ± SEM of three independent experiments, * indicates p<0.05 vs. vehicle. (B) Ultrastructure of megakaryocytes cultured in the presence of vehicle or IFN β from day seven to day fourteen. Inset in the upper panel shows culture-derived platelets observed only in vehicle-treated samples.

Having demonstrated that JUNV infection triggers the production of type I IFN and impairs platelet formation, in the next experiments we examined whether type I IFN was capable of regulating the expression of NF-E2. The results showed that like viral infection, the exposure of purified megakaryocytes to IFN β (Figure 8A) or α (data not shown) resulted in lower NF-E2 expression compared to control samples.

Because NF-E2^−/−^ mice lack circulating platelets and their megakaryocytes show no cytoplasmic platelet formation [33], we analyzed the effect of type I IFN on megakaryocytes at the ultrastructural level. Electron microscopic studies revealed that in contrast with control megakaryocytes, which had a lobulated nucleus and abundant cytoplasm with a mature demarcation membrane system (DMS), in the IFN β-treated cells the DMS was almost absent and lacked organization and platelet territories (Figure 8B). The nucleus looked normal and there was a large cytoplasm, but the contents of the megakaryocyte were not evenly distributed throughout the cytoplasm, including areas with missing granules and organelles. Finally, we observed patches of released platelets that were produced only by megakaryocytes in the control cultures. Interestingly, all these observations resemble the NF-E2^−/−^ megakaryocyte phenotype [33]. IFN α induced similar ultrastructural abnormalities (data not shown).

## Discussion

In this paper, we present evidence that JUNV infects hematopoietic progenitors in a restricted fashion leading to a significant inhibition of thrombopoiesis, including proplatelet formation, platelet release and platelet function.

Previous studies of the effect of viral infection on megakaryocytes derived from CD34^+^ cells showed that human immunodeficiency virus (HIV), human herpesvirus 6 and human cytomegalovirus negatively affected the survival, differentiation and/or maturation of megakaryocyte progenitors [34]–[36]. In contrast, we here demonstrated that JUNV infection had no significant effect on the proliferation, clonogenic ability or maturation of megakaryocyte progenitors, but it induced a profound decrease in proplatelet formation and platelet release. Although impairment of platelet production has been suggested in HIV patients [37], [38], to the best of our knowledge, this is the first time that a viral infection has been shown to selectively affect the two last key steps of thrombopoiesis.

It has recently been demonstrated that TfR1 is the receptor for some new world arenaviruses, including JUNV [27]. Our results show that blocking or down-regulating TfR1 surface expression in CD34^+^ cells significantly reduced both JUNV replication as well as the decrease in platelet formation induced by JUNV infection, indicating that viral infection is a necessary event for the inhibition of platelet formation. Moreover, the fact that UV-irradiated virus had no effect on proplatelet production and platelet release indicates that viral replication is also necessary to hinder thrombopoiesis. These data also implicate TfR1 as the main route of viral entry into hematopoietic progenitor cells. This is particularly relevant considering that it has recently been shown that pathogenic strains can use TfR1-dependent or independent pathways [39]. The observation that JUNV infection up-regulates TfR1 in CD34^+^ cells could represent a viral dissemination strategy at least in hematopoietic bone marrow cells. However, *in vivo* experiments will be required to determine its relevance.

Remarkably, we found that only 5% of total cells were infected, therefore the observed 50% reduction in platelet production appeared to be due to a selective bystander effect from infected cells rather than a direct effect of viral replication on megakaryocyte biology. Moreover, poly(I:C), a synthetic analogue of the dsRNA associated with the replicative cycle of most viruses [28], mimicked the effect of JUNV infection. Although transfected poly(I:C) can be sensed by the retinoic induced gene (RIG)-I-like RNA helicases that stimulate IFN α and β expression, naked poly(I:C) is recognized by TLR3, which predominantly stimulates the expression of NF-kB and IFN β [40]. Therefore, IFN β appeared to be a suitable mediator responsible for the reduced platelet production mediated by JUNV infection of hematopoietic cells. Our results showing: a) the JUNV-dependent induction of IFN β mRNA synthesis, b) the suppressive effect of IFN β on platelet biogenesis, c) the expression of both subunits of the IFN β receptor in purified megakaryocytes, and d) the reversion of the inhibitory effect of JUNV on platelet formation with a neutralizing Ab against IFN β strongly suggest that IFN β is a central mediator in the JUNV-dependent inhibition of platelet release. Similar results were obtained with IFN α (data not shown). This would not be surprising as both IFNs are ligands for the same receptor and IFN α is a downstream product of IFN β production and signaling [30]. Furthermore, both cytokines may be the mediators involved in the bystander effect, and this supports the hypothesis that JUNV and any other diseases that are associated with increased type I IFN levels in the bone marrow milieu may result in thrombocytopenia.

Surprisingly, despite the low number of infected cells, only few were megakaryocytes. Although we have not yet determined the identity of the vWF negative-infected cells, they could be megakaryocytes with low vWF content because it has been recently shown that treatment of megakaryocytes with IFN α inhibited 70% of vWF RNA expression [41].

*In vivo*, JUNV may infect other cells in the bone marrow environment, and CD34^+^ progenitor cells may be exposed to higher levels of type I IFN than produced in our *in vitro* system. Interestingly, we found that while low IFN β concentrations selectively impaired thrombopoiesis, treatment of CD34^+^ cells with higher concentrations also reduced megakaryocyte numbers without modifying the apoptosis rate, suggesting an IFN β-mediated cell cycle arrest. Remarkably, both phenomena were independently described by different groups using recombinant IFN α: while Wang *et al.* demonstrated that IFN α hinders mouse megakaryopoiesis altering proliferation and ploidy [42], Yamane *et al.* found that IFN α decreases platelet production without altering megakaryocyte growth [41]. Although these findings appears to be controversial, our results suggest that the levels of type I IFN in the bone marrow could define the cell fate of megakaryocytes, affecting either their proliferation or their ability to produce platelets.

**Figure 9:**
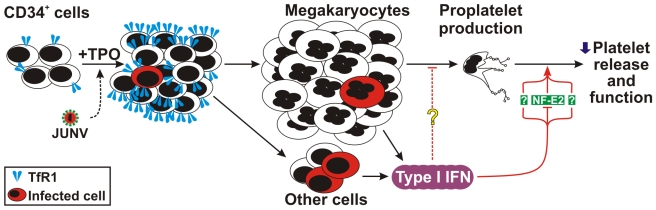
Model of JUNV infection of CD34^+^ cells stimulated with TPO. JUNV induces an increase in the expression of TfR1. A very low proportion of megakaryocytes are infected by JUNV, but the infection triggers type I IFN which impairs proplatelet production, platelet release and platelet function. The concomitant down-regulation of NF-E2 in the megakaryocytic population may be involved in this effect.

Our analysis of the signaling pathways involved in the JUNV-mediated inhibition of platelet production indicated that SOCS-1, SOCS-3, GATA-1, and Src, which are involved in the processes of proliferation, differentiation, and proplatelet formation, respectively [31], [32], [42], were not involved. In contrast, JUNV-infected samples did show lower levels of both NF-E2 mRNA and protein. Interestingly, we also found that type I IFN downregulates NF-E2 in megakaryocytes. As this transcription factor plays a major role in terminal differentiation of megakaryocytes and platelet release [33], it is conceivable that the reduced synthesis of NF-E2 mediated by type I IFN may be one of the molecular pathogenic mechanisms reducing platelet release in JUNV infection (Figure 9). In fact, thrombocytopenia driven by recombinant IFN α has been related to the selective inhibition of cytoplasmic maturation accompanied by downregulation of the expression of the transcription factors NF-E2, GATA-1 and MafG/HPRT [41]. Our ultrastructural studies using IFN β also showed profound alterations in the cytoplasm that could account for the impaired platelet formation. Additionally, we also found a lower number of platelet granules in type I IFN-treated megakaryocytes. Interestingly, NF-E2^−/−^ mice have no circulating platelets and their megakaryocytes present ultrastructural abnormalities that are quite similar to those of our type I IFN-exposed megakaryocytes [33].

As regarding type I IFN's effect on platelet function, elegant studies from Iannacone *et al.* revealed that mice infected with LCMV exhibit a type I IFN-dependent platelet dysfunction that, if associated with thrombocytopenia below a critical threshold, results in severe bleeding and acute anemia [43]. Because they did not observe a direct effect of IFN β on platelet activation, they suggested that the platelet dysfunction could be the result of a direct effect of IFN β on parent megakaryocytes or due to the release from endothelium of platelet inhibitors. Our results showing that platelets derived from JUNV-infected cultures had less P-selectin expression upon thrombin stimulation together with the observation that type I IFN-treated megakaryocytes have almost no dense or alpha granules strongly support the hypothesis that megakaryocyte exposure to type I IFN driven by viral infection of bone marrow cells results not only in a reduced number of platelets but also in the generation of dysfunctional platelets. Nevertheless, more studies are required to further elucidate the hemostatic status and to determine whether similar structural alterations are observed in platelets from VHF patients.

Do the *in vivo* data support our hypothesis regarding the critical role of IFN I in the thrombocytopenia present in VHF patients? High levels of circulating IFN a that correlate with virulence and prognosis has been described in different VHF [44]–[46]. Moreover, during systemic viral infection, bone marrow hematopoietic cells appear to be the most important source of type I IFN [47]. In addition, a direct relationship of thrombocyto/neutropenia driven by type I IFN was demonstrated in LCMV infected mice. In that case, however, the condition was mediated by transient bone marrow aplasia [14]. Overall, these data support a role for type I IFN as a pathogenic factor for the thrombocytopenia observed in VHF patients.

In conclusion, we provide the first evidence linking viral infection of human hematopoietic progenitors with selective inhibition of thrombopoiesis through the type I IFN pathway. Our studies highlight a potential mechanism that leads to thrombocytopenia and bleeding in AHF and other diseases as a result of an increase in the levels of type I IFN in the bone marrow milieu. These data should be of use in the effort to find new therapeutic strategies for the thrombocytopenia that is associated with VHF.

## Materials and Methods

### Ethics statement

This study was conducted according to the principles expressed in the Declaration of Helsinki. The study was approved by the Institutional Review Board of the National Academy of Medicine, Buenos Aires, Argentina. All patients provided written informed consent for the collection of samples and subsequent analysis.

### Reagents

Fluorescein isothiocyanate (FITC)-conjugated monoclonal antibodies (mAbs) against CD34, glycoprotein (GP) IIb (CD41), GP Ib (CD42b), P-selectin (CD62p), phycoerytrin (PE)-conjugated anti-GP IIIa (CD41), unconjugated anti-human HLA-ABC, anti-CD71 Abs, and a fixation/permeabilization kit (BD Cytofix/Cytoperm™) were purchased from BD Biosciences (Franklin Lakes, NJ). FITC-conjugated anti-rabbit immunoglobulins (Igs) and anti-von Willebrand factor (vWF) Abs were obtained from Dako A/S (Glostrup, Denmark). Cy3-conjugated anti-mouse Ig was obtained from Zymed (San Francisco, CA). Recombinant human IFN α_2b_ and rabbit polyclonal anti-human IFN β neutralizing Ab were obtained from Pestka Biomedical Laboratories (Piscataway, NJ). Rabbit polyclonal IgG anti-NF-E2 Ab was purchased from Santa Cruz Biotechnology (Santa Cruz, CA).

Random hexamers, SuperScript III reverse transcriptase and *Trizol* were from Invitrogen. The synthetic analog of dsRNA polyriboinosinic polyribocytidylic acid, poly(I:C) and recombinant human IFN β_1a_ were obtained from InvivoGen (San Diego, CA). The Src family kinase inhibitor 4-amino-5-(4-chlorophenyl)-7-(t-butyl)pyrazolo[3,4-d]pyrimidine (PP2) was obtained from Biomol International LP (Plymouth Meeting, PA). Thrombopoietin (TPO) was obtained from Peprotech (Veracruz, Mexico). All of the other reagents were obtained from Sigma Chemical Co. (St. Louis, MO).

### Isolation of CD34^+^ cells

Isolation of CD34^+^ cells was performed as previously described [48]. Briefly, human umbilical cord blood was collected during normal full-term deliveries and was used within 24 hr. After collection, samples were diluted one to three with PBS and centrifuged to remove platelets. Low density mononuclear cells were prepared by centrifugation of the remaining blood over a Ficoll Hypaque. Cells collected from the interface were washed, and CD34^+^ cells were purified using a magnetic cell-sorting system (Miltenyi Biotec, Bergisch Gladbach, Germany) in accordance with the manufacturer's recommendations. After two Mini-MACS column separations, the purity of the cell suspension was determined by flow cytometry and typically ranged between 95 and 99%. Cell viability was greater than 90%. Fresh CD34^+^ cells were used for each experiment.

### Purification of megakaryocytes

Mature megakaryocytes were purified by immunomagnetic positive selection from TPO-stimulated CD34^+^ cultures on day 12 using anti-CD41 magnetic beads (Miltenyi Biotec) following the manufacturer's instructions. The purity of the final cell suspension was ≥95% and cell viability was greater than 80%.

### Vero-76 cells

Monolayers of Vero-76 cells (CRL-1587, the American Type Culture Collection (ATCC), Manassas, VA) were grown in minimum essential medium (MEM) containing 10% fetal calf serum (FCS) and antibiotics.

### Virus

A virulent strain of JUNV originally isolated from an AHF patient (P3441) was kindly provided by Dr. A. Ambrosio of the *Instituto Nacional de Enfermedades Virales Humanas “Dr. Julio I. Maiztegui”*, Pergamino, Argentina. Virus stocks were grown, identified, and quantified as described previously by using the JUNV-susceptible Vero-76 cell line. Virulence was tested in three-week-old guinea pigs and the median lethal dose, (LD_50_), assayed between 11 and 14 days p.i., was ≤10 PFU. When required, virus was subjected to UV inactivation for 20 min using a 365 nm UV bulb positioned 5 cm over the stock [49].

### Cell infection

CD34^+^ cells (1×10^4^) or a monolayer of Vero-76 cells were inoculated with JUNV at a multiplicity of infection (MOI) of one or the equivalent volume of UV-irradiated virus for 1 hr at 37°C. Mock-infected controls contained supernatants of Vero cells instead of JUNV. After washing, UV-irradiated JUNV-, JUNV- or mock-infected CD34^+^ cells were cultured in Iscove's Modified Dulbecco's Medium (IMDM, HyClone) containing 2 mM glutamine, 5% human serum (obtained by re-calcification of citrated platelet-free plasma), 25 ng/ml TPO and antibiotics (growth medium) at 37°C in a humidified atmosphere with 5% CO_2_ [50]. Fresh TPO (25 ng/ml) was added at day seven. Vero-infected cells were cultured for seven days in MEM containing 2% FCS and antibiotics.

### Detection of JUNV proteins

Cells were washed with PBS (Ca^2+^- and Mg^2+^-free), cytocentrifuged on silanized glasses, fixed with 1% paraformaldehyde (PFA) for 20 min and permeabilized with 0.1% Tween for 10 min. The slides were incubated overnight at 4°C with a pool of specific mAbs against JUNV [22] and a rabbit-anti-human vWF polyclonal Ab to identify megakaryocytes. FITC-conjugated anti-rabbit and Cy3-conjugated anti-mouse Igs were then applied to the PBS-washed slides for 30 min at room temperature (RT). Abs were diluted with PBS containing 5% fetal bovine serum and 5% goat serum as blocking agents. The slides were counterstained with DAPI and examined under an Olympus BX60 microscope equipped with a 100× PlanApo objective (NA 1.4) and a 100-W mercury lamp. Images were acquired with an Applied Imaging Model 4912–5010/0000 charge coupled device (CCD) camera. Electron image acquisition was under the control of Cytovision software.

For flow cytometric studies, samples were washed, fixed with 1% PFA for 20 min, and permeabilized with 0.1% saponin for 10 min. Cells were then incubated for 1 hr at RT with a pool of specific mAbs against JUNV and a rabbit-anti-human vWF polyclonal Ab. After two washes, FITC-conjugated anti-rabbit and Cy3-conjugated anti-mouse Igs were diluted in 0.1% saponin and added to the samples for 30 min and analyzed on a FACSCalibur flow cytometer using CellQuest software (BD Biosciences).

Negative controls were performed using uninfected cells or omitting primary Abs. JUNV-susceptible Vero cells were used as a positive control.

### Evaluation of megakaryocyte development

The levels of CD34, CD41 and CD42b in the TPO-stimulated CD34^+^ cell cultures were measured by immunostaining with saturating concentrations of specific FITC-labeled mAbs or an isotype-matched control after varying numbers of days. After 30 min of incubation, the samples were fixed with 1% PFA and analyzed by flow cytometry.

To evaluate megakaryocyte ploidy, cells were centrifuged for 10 min at 220×*g*, washed and fixed in 70% ethanol at −20°C overnight. Cells were then washed, resuspended in PBS, and incubated for 30 min at RT with saturating concentrations of FITC-conjugated anti-CD41 (or isotype control), 1 µg/ml propidium iodide, and 10 U/ml RNaseA. Cell ploidy was analyzed by flow cytometry.

### Clonogenic progenitor assays

Aliquots of 5×10^3^ CD34^+^ cells were resuspended in collagen-based, serum-free medium containing 50 ng/ml TPO (MegaCult, Stem Cell Technology, Vancouver, Canada) and seeded in double-chamber culture slides. After 12 days, megakaryocyte colonies were detected using an anti-CD41 antibody and an alkaline phosphatase detection system and were then counterstained with Evan's Blue. Two categories of colonies were identified: pure megakaryocyte colonies and mixed megakaryocyte colonies (distinguished by the presence of non-megakaryocyte cells within the same colony). Pure megakaryocyte colonies were scored according to their size: small CFU-megakaryocytes (3–20 cells), medium CFU- megakaryocytes (20–50 cells), and large CFU-megakaryocytes (>50 cells).

### Proplatelet formation (PPF)

PPF was analyzed as previously described with some modifications [51]. Twelve-mm glass coverslips were coated with 100 µg/ml fibrinogen for 2 hr at RT and subsequently blocked with 1% bovine serum albumin for 1 hr. Cells cultured for 12 days were harvested, plated on coated coverslips in 24-well plates (1×10^5^ cells per well) and allowed to adhere for 4 hr at 37°C and 5% CO_2_. PPF was then evaluated by fluorescence microscopy by staining cells with TRITC-conjugated phalloidin. DAPI was used as a counterstain. Proplatelet-forming megakaryocytes were identified as large cells exhibiting long filamentous structures. The extent of PPF was calculated as the percentage of proplatelet-bearing cells by counting 500 cells per treatment.

### Determination of platelet number produced in culture

Platelets were counted using flow cytometry as previously described [25]. Briefly, cells were incubated with a FITC-labeled anti-CD41 mAb for 15 min and fixed with 1% PFA for 20 min. Cells from each culture condition were distributed in the same volume. For each sample, the acquisition rate was 1 µl/sec for 100 sec. Events were collected without gating using a log scale for size (FSC) and intracellular granularity (SSC). An analytical gate was determined based on the scatter properties of normal blood platelets treated similarly to the culture-derived platelets. This gate excluded large contaminating cells and small debris or microparticles. Culture-derived platelets were counted as CD41^+^ events with the same scatter properties as blood platelets.

### P-selectin expression on *in vitro*-produced platelets

P-selectin expression on platelets was determined as previously described with minor modifications [52]. Briefly, culture-derived platelets were isolated by centrifugation at 1000×g in the presence of PGI_2_ to avoid platelet preactivation. The pellet was resuspended in modified Tyrode buffer (134 mM NaCl; 2.9 mM KCl; 0.34 mM Na_2_HPO_4_12H_2_O; 12 mM NaHCO_3_; HEPES 20 mM; MgCl_2_ 1 mM; glucose 5 mM, pH 7.3) and left to rest for 30 min at 37°C. The suspension was stimulated with 1 U/ml thrombin for 10 min at 37°C. After fixation, cells were stained with anti–P-selectin (CD62p)–FITC and anti-CD41-PE mAbs for 30 min and P-selectin expression was measured by flow cytometry using the characteristic forward and side scatter pattern of normal blood platelets treated similarly.

### Expression of TfR1 and NF-E2

To determine the level of TfR1 expression on TPO-stimulated CD34^+^ cell membranes, cells were incubated with a FITC-conjugated anti-CD71 or an isotype-matched control mAb at the indicated days p.i. Samples were washed, fixed, and analyzed by flow cytometry.

CD34-derived megakaryocytes were tested for NF-E2 expression using a double-labeling technique (NF-E2/CD41). Cells were immunostained with a PE-conjugated anti-CD41 mAb or an isotype-matched control and fixed with 1% PFA at 4°C. After washing with PBS containing 0.1% saponin, cells were incubated first for 30 min in the same buffer with anti-NF-E2 polyclonal Ab at 4°C and then with FITC-conjugated swine anti-rabbit Igs. Cells were analyzed by flow cytometry. Non-specific fluorescence was assessed using rabbit serum instead of primary Ab. In selected experiments, NF-E2 expression was determined in purified mature megakaryocytes treated with type I IFN using the anti-NF-E2 polyclonal Ab (or rabbit serum) followed by FITC-conjugated swine anti-rabbit Igs.

### RNA isolation and semi-quantitative RT-PCR

Total RNA was isolated from cell pellets using *Trizol* as recommended by the manufacturer. cDNA was synthesized from 20 ng of total RNA using 15 mM of random hexamers and SuperScript III reverse transcriptase according to the manufacturer's instructions. The cDNA samples were diluted 10-fold, and the PCR reaction was conducted at the annealing temperature of 55°C. All reactions were confirmed to be within the linear range of amplification. The primer sequences and sizes of the amplified fragments are included in Table 1.

**Table 1:** Primers used for RT-PCR (5′→3′).

Gene	Fragment size (bp)	Forward	Reverse
JUNV	640	CGCACCGGGGATCCTAGGC	GGCATWGANCCAAACTGATT
IFN α	274	TCCATGAGATGATCCAGCAG	ATTTCTGCTCTGACAACCTCCC
IFN β	286	GCTCTCCTGTTGTGCTTCTCCAC	CAATAGTCTCATTCCAGCCAGTGC
IFNAR1	326	GTGATACACATCTCTCCTGG	GTATAATCCCATTTAAGAACATAG
IFNAR2	394	GAGTAAACCAGAAGATTTGAAG	CGTGTTTGGAATTAACTTGTC
NF-E2	255	CAGTAGGATGTCCCCGTGTC	TAAGGTGGTGGAGGAAGTGG
GATA-1	613	ATTGTCAGTAAACGGGCAGG	GCTTTGAAGGTTCAAGCCAG
SOCS-1	562	AGAGCTTCGACTGCCTCTT	AGGTAGGAGGTGCGAGTTCA
SOCS-3	554	CTCAAGACCTTCAGCTCCAA	TTCTCATAGGAGTCCAGGTG
GAPDH	996	GTGAAGGTCGGAGTCAACG	TCCTTGGAGGCCATGTGGGCCCT

The following symbols were used for mixed bases: W, A:T; N, A:C:G:T.

### Thin-section electron microscopy

Megakaryocytes were fixed with 1.5% glutaraldehyde in 0.1 M cacodylate buffer, pH 7.4, for eight hr. Cells were dehydrated through a series of alcohols, infiltrated with propylene oxide, and embedded in epoxy resin in an inverted beam capsule. Ultrathin sections were stained with uranyl acetate and lead citrate and examined with a Tecnai G2 Spirit BioTWIN transmission electron microscope (Hillsboro, OR) at an accelerating voltage of 80 kV, and images were recorded with an AMT 2k CCD camera (Danvers, MA).

### Statistical analysis

All results are expressed as the means ± SEM. Student's paired *t* test was used to determine the significance of differences between means, and *p* values lower than 0.05 were considered to be statistically significant. When multiple groups were compared, one-way analysis of variance (ANOVA) followed by the Newman-Keuls procedure was used to determine significant differences between groups.
